# Microbial Community Distribution in Low Permeability Reservoirs and Their Positive Impact on Enhanced Oil Recovery

**DOI:** 10.3390/microorganisms13061230

**Published:** 2025-05-27

**Authors:** Jin Pang, Tongtong Wu, Xinan Yu, Chunxi Zhou, Jiaao Gao, Haotian Chen

**Affiliations:** School of Petroleum and Gas Engineering, Chongqing University of Science and Technology, Chongqing 401331, China; 2023201095@cqust.edu.cn (T.W.); 2010019@cqust.edu.cn (X.Y.); 2023201092@cqust.edu.cn (C.Z.); 2024201059@cqust.edu.cn (J.G.); 2024201071@cqust.edu.cn (H.C.)

**Keywords:** low permeability reservoir, microbial enhanced oil recovery, microbial community, oil recovery efficiency, high-throughput genomics, metabolic products

## Abstract

Low permeability oil reservoirs hold an important position in the global oil resource reserves. They boast abundant reserves and serve as one of the crucial sources for crude oil reserve replacement in China and even the world. The mechanisms for improving the oil recovery rate in high-oil-bearing reservoirs include improving fluid properties, enhancing displacement efficiency, etc. However, their development is quite challenging, requiring continuous exploration and innovation in development technologies. This study addresses the unclear distribution patterns of microbial communities and the incomplete understanding of microbial enhanced oil recovery (MEOR) mechanisms in low permeability reservoirs. Utilizing high-throughput genomics and functional gene analysis techniques, combined with laboratory and field data, the study investigates the distribution and growth patterns of microbial communities in a low permeability reservoir, exemplified by the S169 block. Additionally, the potential of MEOR to enhance oil recovery and its underlying mechanisms are explored. The results indicate that microbial communities in low permeability reservoirs exhibit strong heterogeneity, with their distribution closely correlated to geological factors such as reservoir permeability and porosity. The diversity of microbial communities is positively correlated with oil recovery efficiency, and highly active microbial populations promote the production of metabolites that enhance oil recovery. The metabolic products of microorganisms help reduce the interfacial tension between oil and water, improve the fluidity of oil, and enhance the recovery rate. In addition, the structural changes in microbial communities are closely related to factors such as the permeability and porosity of reservoirs. This study provides a theoretical basis for the optimization of microbial enhanced oil recovery (MEOR) technology.

## 1. Introduction

Low permeability reservoirs refer to reservoirs with relatively low permeability, typically defined as reservoirs with a matrix permeability of less than 50 mD [1,2,3]. The reservoir rocks of low permeability reservoirs are usually tight, characterized by small pore throats, low porosity, and poor permeability [4,5]. Their micro-pore structures are complex, with small dominant flow throat radii and a low percentage of movable fluids. These reservoirs exhibit strong heterogeneity, with significant permeability anisotropy [6,7]. The permeability varies greatly in different directions, and microfractures are often well-developed. Due to the low permeability, the flow resistance of fluids within the reservoir is high, making conventional development methods ineffective for large-scale exploitation [8,9]. During the development process, there is a starting pressure gradient, which makes the seepage characteristics of the fluids difficult to describe and prone to issues such as water flooding and water coning [10,11]. As global oil resources gradually deplete, low permeability reservoirs are becoming a crucial focus for petroleum exploration and development. In particular, during high water cut stages in oilfields, improving recovery rates while reducing costs has become a pressing issue [12].

Microbial enhanced oil recovery (MEOR), a cost-effective and environmentally friendly technique, has been increasingly applied to the development of reservoirs [13,14]. The basic principle of MEOR is to inject microorganisms or their metabolic products into the reservoir, utilizing bio-surfactants, organic acids, and biogas to alter the fluid properties of the reservoir, reduce the oil-water interfacial tension, and thereby improve oil recovery [15,16].

The distribution and growth patterns of microbial communities are key factors influencing MEOR effectiveness [17]. Microbial communities in low permeability reservoirs typically exhibit strong heterogeneity, which is closely related to geological factors such as permeability, porosity, and other reservoir characteristics [18,19]. Studies have shown that microbial communities in low permeability reservoirs mainly consist of bacteria, archaea, and methanogens, all of which can survive and function under harsh reservoir conditions [20,21,22].

With the advancement of high-throughput genomics and functional gene analysis technologies, researchers can now more accurately reveal the dynamic changes in microbial communities and their impact on oil recovery efficiency [23,24]. For example, the diversity of microbial communities is positively correlated with oil recovery efficiency [25]. Furthermore, different microbial populations contribute differently to recovery rates, with the presence of highly active microbial groups typically associated with higher rates of metabolic product production and improved oil recovery outcomes [26,27].

These research findings provide a theoretical basis for optimizing microbial enhanced oil recovery (MEOR) technology and offer a new perspective for the development of low permeability reservoirs [28,29]. However, there are still some deficiencies in the current research on microbial oil recovery in low permeability reservoirs. Firstly, the relationship between microbial community heterogeneity and reservoir geological conditions has not been fully clarified, and there is a lack of systematic research on its distribution patterns. Secondly, although microbial metabolic products have played a certain role in enhancing oil recovery, the specific metabolic products and the synergistic mechanism among different microbial populations remain to be further elucidated. Finally, although laboratory studies have demonstrated the application potential of microbial enhanced oil recovery technology, it still faces challenges in terms of field application and long-term stability [30,31,32].

This study takes the S169 block as a case and combines field and laboratory experiments to innovatively explore the distribution patterns and growth laws of microbial communities in low permeability reservoirs, as well as their impacts on crude oil recovery mechanisms. The objectives include verifying whether microbial activated water flooding has a promoting effect on reservoirs with an average permeability far lower than the recommended value of 50 millidarcies (mD). It aims to reveal the distribution patterns of microbial communities under complex geological conditions, analyze the relationship between microbial growth and crude oil recovery, and optimize the microbial community structure to improve the oil recovery rate of reservoirs. This study is intended to provide theoretical guidance for optimizing the microbial enhanced oil recovery (MEOR) technology and promote its industrial application.

## 2. Materials and Methods

### 2.1. Reservoir Characteristics of the Experimental Area

The experimental samples used in this study were collected from the S169 block. Located in Ansai County, Yan’an City, Shaanxi Province, China, the S169 block is a typical structural-lithologic reservoir (approximately 36°48′ north latitude and 109°12′ east longitude). This block has been undergoing rolling development through the revitalization of old wells. The currently utilized oil-bearing area is 12.51 km^2^, with an oil geological reserve of 809.32 × 10^4^ tons. The main development targets are the Yan82, Yan91, and Yan92 formations. The sedimentary microfacies of the reservoir is characterized by delta front underwater distributary channels, with an average oil layer thickness of 7.6 m, porosity of 18.0%, and permeability ranging from 0.34 to 132.8 mD, with an average of 19.0 mD, indicating strong heterogeneity. The field has been under development since 2001, with water injection implemented in 2003. An irregular inverse seven-point well pattern is used, with a well density of 14.6 wells/km^2^. The average recoverable reserves per well are approximately 1.62 × 10^4^ tons, with a daily liquid production of 312 m^3^/d and daily oil production of 85 t/d. The comprehensive water cut is 71.1%. Current challenges include low well production during the high water-cut stage, with 67% of wells having daily production lower than 1.5 t/d and 55% of wells having a water cut exceeding 60%. Additionally, the vertical permeability heterogeneity is significant, making waterflooding adjustment difficult, with a predicted waterflood recovery factor of only 27.5%.

### 2.2. Experimental Materials

(1) Enrichment Pond Water Samples

Water samples for this study were collected from the microbial enrichment ponds at the water injection station in the S169 block, used for microbial concentration and metabolite concentration analysis.

The high-efficiency oil-displacing engineered bacterial strain CQM-3 used in the propagation tank was a Gram-positive spore-forming bacillus that was developed through targeted screening for efficient oil recovery. It exhibited rod-shaped or oval morphology and could form spores to enhance environmental adaptability. The strain grew optimally at temperatures of 30–55 °C and a pH range of 6.5–7.5, demonstrating strong reservoir adaptability. CQM-3 utilized long-chain alkanes in crude oil as its primary carbon source [33]. Under optimal conditions, the viable cell concentration could exceed 1 × 10^8^ CFU/mL, with a crude oil degradation rate of over 85%. It preferentially degraded components in the C15–C30 range and could tolerate salt concentrations of up to 15%. This strain improved oil recovery efficiency through multiple synergistic mechanisms, including reducing the interfacial tension between oil and water to approximately 10^−2^ mN/m via biosurfactants (such as lipopeptides and glycolipids) in its metabolic byproducts; secreting short-chain organic acids (C2–C6) to dissolve carbonate rocks and enlarge pore throat diameters; producing gases (mainly CO_2_ and CH_4_) under anaerobic conditions to increase formation pressure and reduce crude oil viscosity; and forming biofilms to selectively plug high-permeability channels, thereby achieving fluid profile control [34,35].

For industrial production, a two-stage fermentation process was employed, with strict control of dissolved oxygen (3–5 mg/L) and pH (6.8 ± 0.2) to ensure the quality of the bacterial cells. The final product was characterized by a high viable cell count, a spore formation rate exceeding 90%, and low endotoxin content. After sterilization through a 0.22 μm membrane filter, it could be stably stored at 4 °C for up to 6 months [36].

Two batches of enrichment pond water samples were collected for this study. The first batch was collected on 3 September 2024, from three enrichment ponds, while the second batch was collected on 18 October 2024, from the same three ponds.

(2) Wellhead Produced Water Samples

Water samples for functional gene quantification and high-throughput sequencing analysis were collected from different oil production wells in the S169 block.

Microbial culture liquid, cultivated in the surface enrichment ponds, was injected into water injection wells and flowed through the reservoir by percolation, eventually reaching the oil wells. Wellhead liquid was collected from various oil wells, and after sedimentation and centrifugation, the separated water samples were extracted for analysis.

Two batches of wellhead liquid samples were collected for this experiment. The first batch was collected on 3 September 2024, from 24 oil wells, and the second batch was collected on 18 October 2024, from 15 oil wells.

### 2.3. Experimental Methods

Four experiments were designed according to the research objectives.

(1) Microbial Concentration Detection:

The microbial concentration in the samples was determined using the plate count method (The substrates are tryptone, yeast extract, glucose, agar, and distilled water) [37,38]. The experimental procedure is as follows: First, 25 mL of the sample was aspirated with a sterile pipette and transferred into a sterile conical flask containing 225 mL of phosphate buffer solution or physiological saline. The mixture was then thoroughly mixed to prepare a 1:10 sample homogenate. The homogenate was incubated at 36 ± 1 °C for 48 ± 2 h. Next, 1 mL of the 1:10 sample homogenate was aspirated with a 1-mL sterile pipette and injected into a sterile test tube containing 9 mL of diluent. The mixture was thoroughly mixed to prepare a 1:100 sample homogenate. This dilution process was continued, with the pipette or pipette tip being changed with each dilution. Two to three appropriate dilutions of the sample homogenate were selected. Fifty microliters of each dilution were transferred into separate sterile culture plates. The operation was performed near an alcohol lamp. When the temperature of the agar dropped to 46 °C, it was poured into the culture plates to a depth of 1/3 and thoroughly mixed. After the agar solidified, the plates were inverted and incubated at 36 ± 1 °C for 48 ± 2 h. If the sample might contain diffuse-growing colonies, the surface of the solidified agar was covered with approximately 4 mL of a thin layer of agar medium after solidification, and the plates were then inverted again. The colonies were observed with the naked eye. If necessary, a magnifying glass or a colony counter was used to record the dilution factor and the number of colonies, expressed in CFU [39,40].

(2) Microbial Metabolite Monitoring:

This experiment utilized analytical instruments such as gas chromatography (GC), gas chromatography-mass spectrometry (GC-MS) [41] (GCMS-QP2020 NX, Shimadzu Corporation, Kyoto, Japan: the type of chromatographic column is HP-5MS, with dimensions of 30 m × 0.25 mm × 0.25 μm in terms of length/internal diameter/film thickness), and a Fourier transform infrared spectrometer [42,43,44] (FI-RXF300V, Beijing Zolix Instruments Co., Ltd., Beijing, China: a vacuum-type Fourier transform infrared spectrometer with a full vacuum optical design, spectral range of 6000–50 cm^−1^, and spectral resolution of ≤0.25 cm^−1^) to detect microbial metabolites, including organic acids, alcohols, ketones, aldehydes, ethers, esters, and biogas.

Short-chain organic acids (mainly C1–C5 small molecule organic acids) were tested directly by gas chromatography. The retention time of the standard (short-chain organic acids, analytical grade) was used for qualitative analysis. A short-chain organic acid that was not present in a certain sample was selected as the internal standard, and the quantitative analysis of short-chain organic acids was carried out by the peak area integration method of the GC spectrum.

Long-chain organic acids (≥C6) were processed by centrifugation and filtration of the bacterial liquid sample, followed by derivatization with a derivatization reagent. The sample was then analyzed by gas chromatography-mass spectrometry (GC-MS). The qualitative analysis was performed by searching the mass spectral library NIST98, and the quantitative analysis was carried out using hexadecanoic acid and octadecanoic acid as external standards. Alcohols and phenols were extracted from the sample with dichloromethane. Acetic anhydride was added to the aqueous phase for reaction, followed by extraction with toluene. The organic phase was analyzed by GC-MS. Aldehydes and ketones, which are compounds containing carbonyl groups, reacted with derivatization reagents in an acidic medium to form derivatives. After extraction with solvents such as carbon disulfide, the samples were analyzed by gas chromatography-mass spectrometry. The qualitative analysis was performed by searching the mass spectral library NIST98, and the quantitative analysis was carried out using acetaldehyde as an external standard.

Lipids were processed by adding HCl to the bacterial liquid sample to adjust the pH value. The lipids in the bacterial liquid were extracted with chloroform/methanol (volume ratio 2:1). The mixture was stirred and re-extracted. The organic phases were combined, and the solvent was evaporated to obtain the crude product. The crude product was dissolved in chloroform and subjected to silica gel thin-layer chromatography. Different color-developing agents were used to preliminarily determine the type of lipid compounds. The crude product was pressed into a KBr pellet, and its infrared spectrum was measured in the range of 4000–400 cm^−1^. The sample was analyzed by chromatography-mass spectrometry. The qualitative analysis was performed by searching the mass spectral library NIST98, and the quantitative analysis was carried out using phthalic anhydride as an external standard. Biogas was analyzed by cultivating oil-displacing bacteria in serum bottles until the growth reached a stable phase. The gas inside the bottle was then drawn and analyzed by gas chromatography.

(3) Functional Gene Quantification:

Quantitative PCR (qPCR) was used to detect the expression of functional genes in the microbial communities in low permeability reservoirs [45,46]. Target genes, including 16S rRNA (total bacteria), Arch (total archaea), and mcrA (total methanogens), were quantified. The experimental process involved the EDC-810 PCR Instrument (Beijing Eastwin Biotechnology Co., Ltd., Beijing, China) sample collection, DNA extraction, primer design, standard curve construction, and qPCR amplification. High-precision instruments such as vortex shakers, clean benches, high-speed centrifuges, and NanoDrop 5000 (Thermo Fisher Scientific, Wilmington, Delaware, USA) were used in this process. The purpose of this experiment was to explore the functional characteristics of microbial communities and provide theoretical support and data for the application of MEOR in low permeability reservoirs. The primers used for gene detection are shown in Table 1.

(4) High-Throughput Sequencing Analysis:

This experiment utilized high-throughput sequencing technology to analyze microbial diversity [47,48]. The process involved total DNA extraction from the samples, PCR amplification, library construction, and quality control, followed by high-throughput sequencing. The raw data obtained were subjected to quality control, assembly, and denoising processes to generate high-quality sequences. Subsequently, feature sequence analysis (OTU/ASV) was conducted for species classification and community structure analysis. Alpha diversity and beta diversity analyses were performed to reveal the differences between samples and their species distribution. The purpose of this experiment was to elucidate the microbial community composition and diversity in different samples, providing foundational data for subsequent functional predictions and environmental factor analyses.

## 3. Results

### 3.1. Microbial Concentration

**Figure 1:**
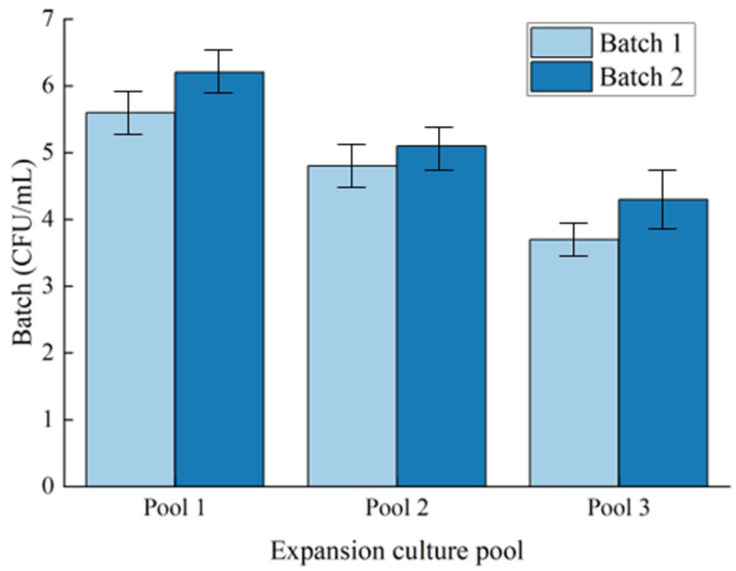
Changes in microbial concentration across different experimental batches.

In the detection of microbial concentrations in two batches, the microbial concentrations in all three propagation pools increased (Figure 1), with increases of 0.6 × 10^6^ CFU/mL, 0.3 × 10^6^ CFU/mL, and 0.6 × 10^6^ CFU/mL, respectively. The growth rates were 10.71%, 6.25%, and 16.22%, respectively.

These data indicate that the microbial community in the expansion tank exhibits enhanced reproductive activity during the subsequent culture process. There is a positive correlation between microbial concentration and culture time (r = 0.85, *p* < 0.05), indicating that the increase in microbial concentration is not a random fluctuation.

### 3.2. Metabolite Concentrations

(1) Surfactants (Lipids)

**Figure 2:**
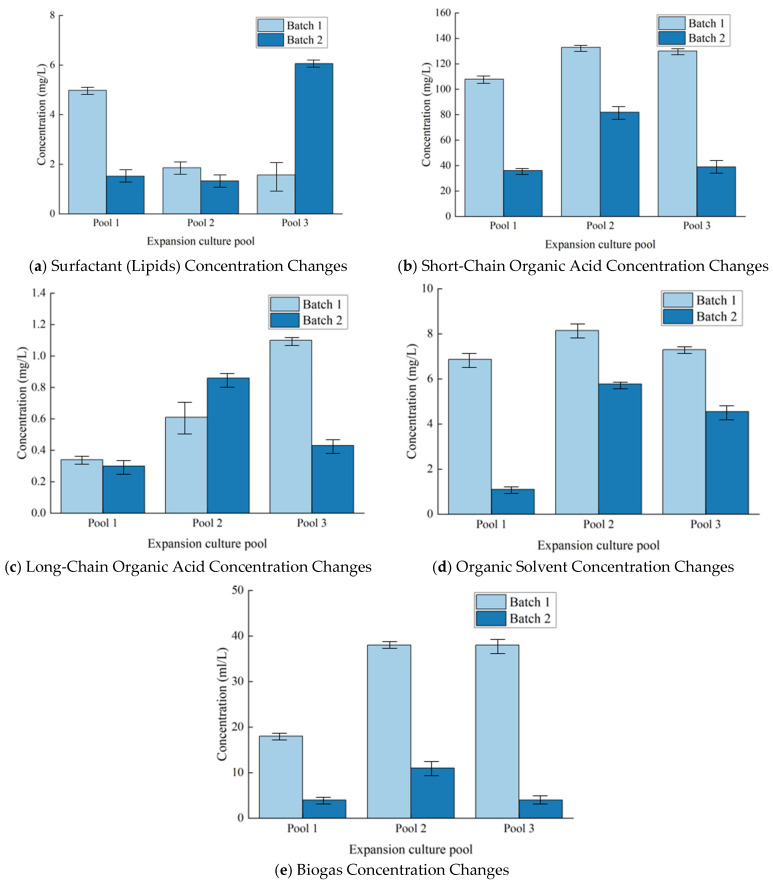
Changes in microbial metabolite concentrations across different experimental batches.

In the first experimental batch, the lipid concentrations in Pool 1, Pool 2, and Pool 3 were 4.98 mg/L, 1.86 mg/L, and 1.57 mg/L, respectively. In the second batch, the lipid concentrations were 1.52 mg/L, 1.33 mg/L, and 6.06 mg/L, respectively (Figure 2a, Table 2).

From the data, it can be observed that the lipid concentration in Pool 3 increased in the second batch (from 1.57 mg/L to 6.06 mg/L).

From the data results, it can be observed that the lipid concentration in Pool 3 increased in the second batch (+287%, *p* < 0.05). This change suggests that the metabolic activity of the microorganisms in this pool may have been enhanced, which is speculated to be closely related to the improved adaptability of the microbial community or the optimization of external conditions. In contrast, the lipid concentrations in Pool 1 and Pool 2 slightly decreased in the second batch (1.52 mg/L and 1.33 mg/L, respectively), which may be associated with a shift in the microbial metabolic pathways.

(2) Short-Chain Organic Acids

The total concentration of short-chain organic acids (such as formic acid, acetic acid, propionic acid, etc.) in the first batch was 108 mg/L for Pool 1, 133 mg/L for Pool 2, and 130 mg/L for Pool 3. In the second batch, the concentrations were 36.30 mg/L for Pool 1, 82.86 mg/L for Pool 2, and 39.43 mg/L for Pool 3 (Figure 2b, Table 2).

In the second batch, the overall concentration of short-chain organic acids decreased, especially in Pool 1 (*p* = 0.001) and Pool 3 (*p* < 0.001). Short-chain organic acids may have been converted into other metabolic byproducts such as long-chain organic acids or biogas. In contrast, the concentration of short-chain organic acids in expansion Pool 2 (*p* = 0.012) remained relatively high between the two batches, indicating that the microbial metabolic processes in this pool were more inclined toward the accumulation of short-chain organic acids.

(3) Long-Chain Organic Acids

The total concentration of long-chain organic acids (such as hexadecanoic acid, octadecanoic acid, etc.) in the first batch was 0.34 mg/L for Pool 1, 0.61 mg/L for Pool 2, and 1.10 mg/L for Pool 3. In the second batch, the concentrations were 0.30 mg/L for Pool 1, 0.86 mg/L for Pool 2, and 0.43 mg/L for Pool 3 (Figure 2c, Table 2).

In the second batch, the concentration of long-chain organic acids in expansion Pool 2 increased (*p* = 0.023), reflecting an enhanced conversion process from short-chain organic acids to long-chain organic acids. In contrast, the concentration of long-chain organic acids in Pool 3 decreased in the second batch (*p* = 0.007), which may be related to a reduction in conversion efficiency or further decomposition of long-chain products. Correlation analysis was conducted between the changes in long-chain organic acid concentration and microbial concentration in each pool. The results showed that the increase in long-chain organic acid concentration in Pool 2 was positively correlated with the increase in microbial concentration (r = 0.78, *p* = 0.045), while the decrease in long-chain organic acid concentration in Pool 3 was not significantly correlated with the increase in microbial concentration (r = −0.32, *p* = 0.21). This indicates that the metabolic changes in Pool 2 may be closely related to the increase in microbial population, while the changes in Pool 3 may be influenced by other factors.

(4) Organic Solvents

The total concentration of organic solvents (such as alcohols, ketones, aldehydes, and ethers) in the first batch was 6.87 mg/L for Pool 1, 8.15 mg/L for Pool 2, and 7.30 mg/L for Pool 3. In the second batch, the concentrations were 1.10 mg/L for Pool 1, 5.77 mg/L for Pool 2, and 4.55 mg/L for Pool 3 (Figure 2d, Table 2).

In the second batch, the concentration of organic solvent in Pool 1 decreased by 84%, indicating that the organic solvent may have been converted into other metabolites. The difference in organic solvent concentration between the two batches in Pool 1 was statistically significant (*p* < 0.01), suggesting that this change was not a random fluctuation but a direct result of altered microbial metabolic activity. In contrast, the changes in organic solvent concentration in Pools 2 and 3 were relatively small between the two batches. The concentration in Pool 2 decreased from 8.15 mg/L to 5.77 mg/L, and the concentration in Pool 3 decreased from 7.30 mg/L to 4.55 mg/L. These changes did not reach the significance level (*p* > 0.05), indicating that the microorganisms in these two pools were still actively carrying out organic solvent-related metabolism, and the metabolic pathways might be relatively stable.

(5) Biogas

The total volume of biogas in the first batch was 18 mL/L for Pool 1, 38 mL/L for Pool 2, and 38 mL/L for Pool 3. In the second batch, the biogas volumes were 4 mL/L for Pool 1, 11 mL/L for Pool 2, and 4 mL/L for Pool 3 (Figure 2e, Table 2).

The results of the statistical analysis show that the difference in the total amount of biogas between the first batch and the second batch is clear (*p* < 0.05), indicating that this change is not accidental. In addition, there is a certain negative correlation between the total amount of biogas and the microbial concentration (r = −0.76, *p* < 0.05), which may suggest that the metabolic activity of microorganisms is limited, resulting in a decrease in gas production.

### 3.3. Quantitative Analysis of Functional Genes

The gene abundance data from the two batches of functional gene quantitative analysis were extracted. A comparison chart of the abundance of the 16S rRNA gene, Arch archaeal gene, and mcrA methane-producing bacterial gene was created for analysis.

(1) 16S rRNA Gene Abundance

**Figure 3:**
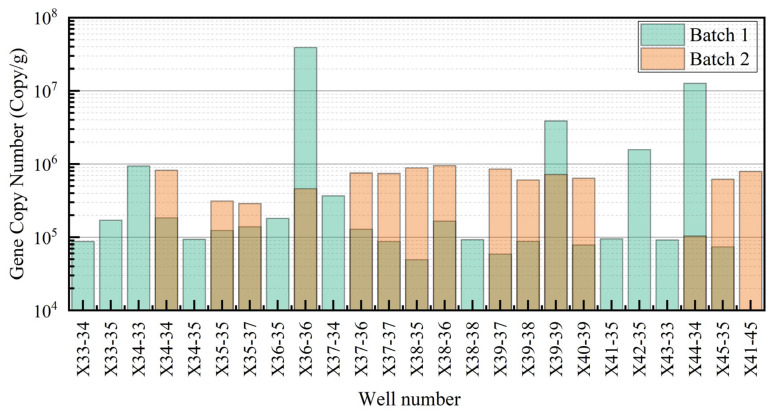
Comparison of 16S rRNA gene abundance in different experimental batches.

In the first batch of samples, the abundance of the 16S rRNA gene was generally high, with an average abundance of 2.51 × 10^6^ copies/g, a standard deviation of 8.18 × 10^6^ copies/g, a minimum of 4.92 × 10^4^ copies/g, and a maximum of 3.89 × 10^7^ copies/g, showing fluctuations. Notably, certain wells (e.g., X36-36 and X44-34) exhibited higher gene abundance (Figure 3).

In the second batch, the gene abundance was more uniform, with an average abundance of 6.37 × 10^5^ copies/g, a standard deviation of 2.45 × 10^5^ copies/g, a minimum of 1.04 × 10^5^ copies/g, and a maximum of 9.51 × 10^5^ copies/g. The peak abundance values were less frequent, possibly indicating overall suppression of gene amplification or ecological adjustment (Figure 3).

(2) Arch (Archaeal) Gene Abundance

**Figure 4:**
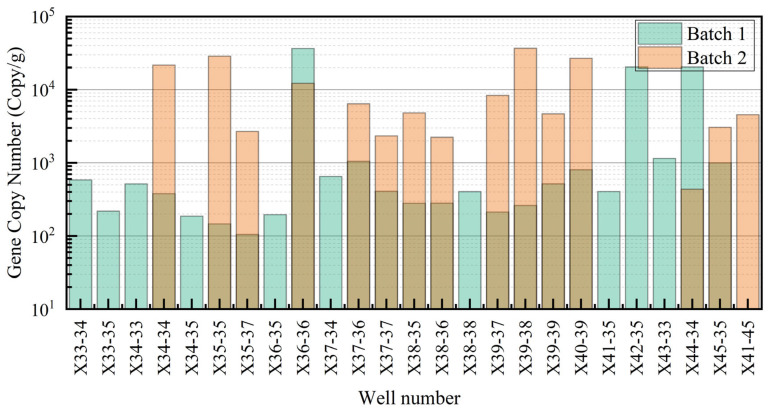
Comparison of archaeal gene abundance in different experimental batches.

In the first batch samples, the abundance of archaeal genes was low, with an average abundance of 1.17 × 10^5^ copies/g, a standard deviation of 4.29 × 10^5^ copies/g, a minimum value of 9.65 × 10^3^ copies/g, and a maximum value of 2.11 × 10^6^ copies/g. Despite the generally low abundance, some wells (such as X36-36 and X42-35) showed higher gene abundance (Figure 4).

In the second batch samples, the archaeal gene abundance was slightly higher than in the first batch, with an average abundance of 2.75 × 10^4^ copies/g, a standard deviation of 4.42 × 10^3^ copies/g, a minimum value of 2.03 × 10^4^ copies/g, and a maximum value of 3.52 × 10^4^ copies/g. However, the overall abundance remained relatively low, with a small range of variation. (Figure 4).

(3) mcrA (Methanogen) Gene Abundance

**Figure 5:**
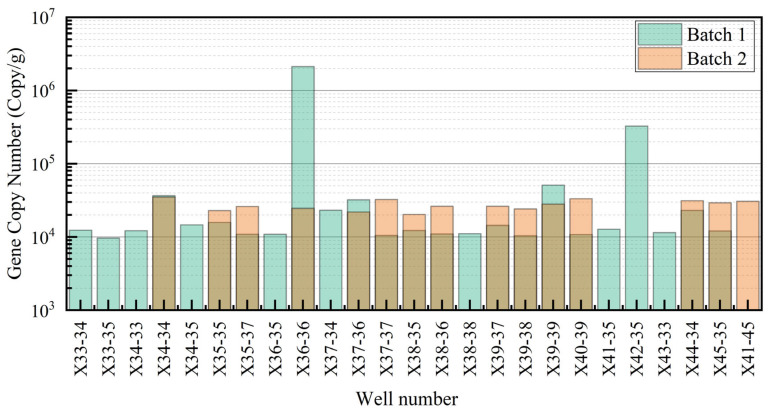
Comparison of mcrA methanogen gene abundance in different experimental batches.

In the first batch of samples, the average mcrA gene abundance was 3623.42 copies/g, with a standard deviation of 8965.19 copies/g, a minimum value of 105 copies/g, and a maximum value of 36,400 copies/g. The mcrA abundance showed an increase in a few wells (such as X36-36), while the rest of the wells had relatively low abundance (Figure 5).

In the second batch of samples, the average abundance was 11,029 copies/g, with a standard deviation of 11,575 copies/g, a minimum value of 435 copies/g, and a maximum value of 36,600 copies/g. The mcrA abundance tended to stabilize in most wells, but a few wells (such as X36-36) showed changes (Figure 5).

### 3.4. Microbial Community Composition

(1) Distribution Characteristics of Bacterial and Archaeal Communities

Analysis of high-throughput sequencing data from different well points and batches revealed differences in the distribution characteristics of bacterial and archaeal communities, which were closely related to the geological characteristics of the well points, such as porosity and permeability. In the first batch of data, bacterial community abundance varied greatly between well points, particularly in well points with higher abundance of *Proteobacteria* and *Actinobacteria*, which are likely involved in the degradation of organic matter in the reservoir and could affect the permeability of the oil layer. In contrast, the abundance of archaea was generally low, especially at some well points where archaeal abundance was notably reduced. This phenomenon may be related to the anaerobic environment and the degree of organic matter degradation in these areas.

Compared to the first batch, the bacterial community abundance in the second batch generally decreased, with a particularly notable reduction in *Proteobacteria* abundance. However, archaeal community abundance increased, especially in deeper well points, where the abundance of methane-producing bacteria (*Methanobacteriaceae*) rose. This change suggests a close relationship between methane production during the water flooding process and the proliferation of archaea, reflecting a synergy between the enhanced archaeal community and the gas generation demand of the reservoir.

Further analysis showed that the porosity and permeability of the well points directly influenced the abundance and distribution of bacterial and archaeal communities. Specifically, in well points with higher permeability, bacterial community abundance was generally higher, while archaeal abundance was lower. In certain well points (“New 38-35”), both bacterial and archaeal communities were active, regardless of the batch. This indicates that microbial community activation may promote microbial growth and metabolism during water flooding.

(2) Changes in Bacterial and Archaeal Communities

During the water flooding process in different batches, bacterial and archaeal communities exhibited changes, reflecting the complex relationship between environmental factors and microbial activity.

Changes in Bacterial Communities:

In the first batch, the bacterial community was predominantly dominated by *Proteobacteria* and *Actinobacteria*, which are involved in the degradation of organic matter in the reservoir, thereby improving the permeability of the oil layer. However, in the second batch, bacterial community abundance decreased, and its diversity also reduced. This change may be closely related to environmental changes during water flooding (such as temperature and pressure). Environmental fluctuations likely inhibited the growth of some bacterial communities, leading to a decline in abundance, indicating that environmental variations during water flooding influence microbial communities.

Changes in Archaeal Communities:

In the first batch, the abundance of archaea was relatively low, and methane-producing bacteria were the dominant group, indicating low archaeal activity during the water flooding process. In contrast, in the second batch, the activity of archaeal communities increased, especially in some well points (“New 34-34” and “New 35-35”), where the abundance of methane-producing bacteria rose. This change reflects the enhancement of the archaeal community, which is closely related to methane production needs during water flooding, potentially supporting gas generation in the reservoir.

(3) Relationship Between Microbial Communities and Reservoir Characteristics

**Figure 6:**
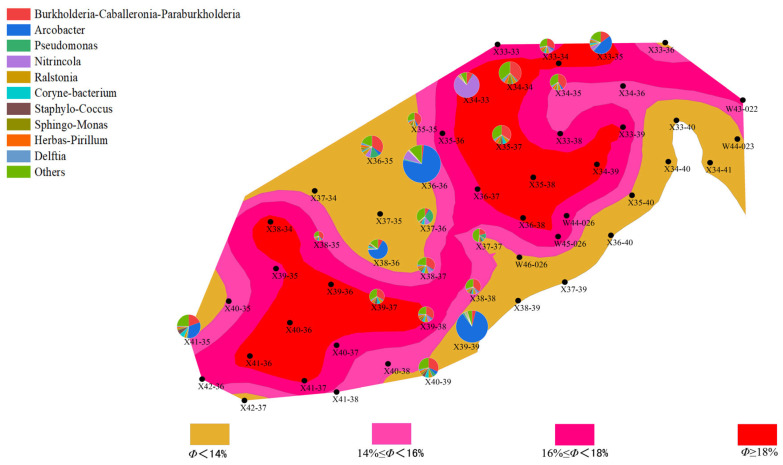
Overlay diagram of reservoir porosity and bacteria distribution in the first batch of detected samples.

**Figure 7:**
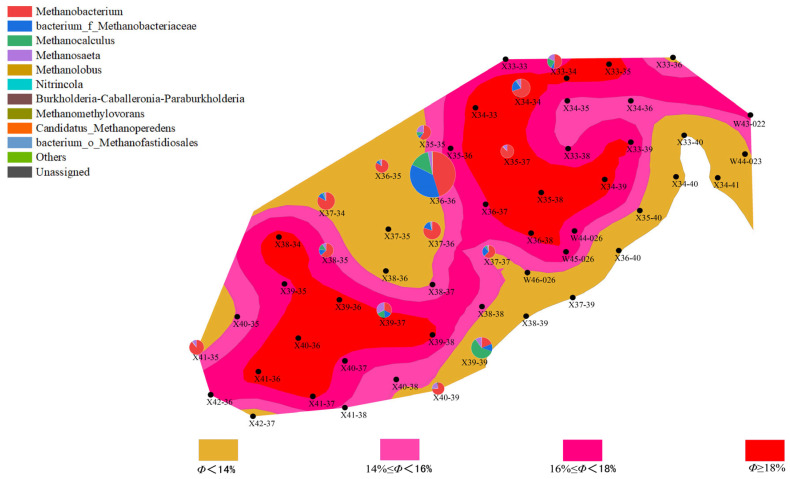
Overlay diagram of reservoir porosity and archaea distribution in the first batch of detected samples.

**Figure 8:**
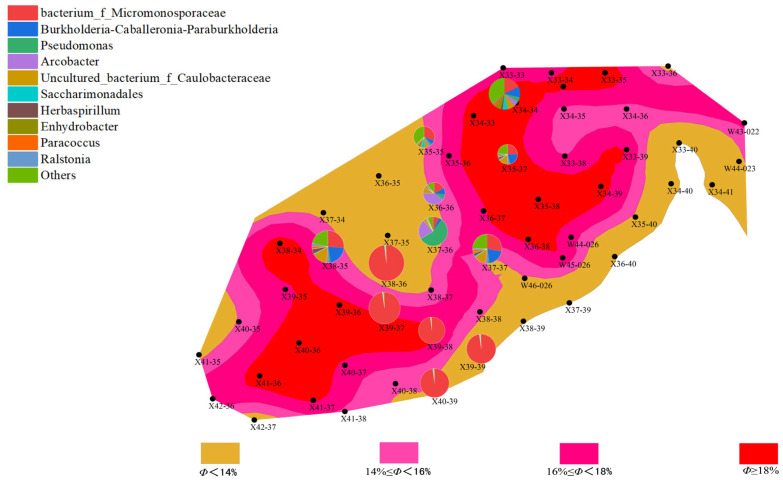
Overlay diagram of reservoir porosity and bacteria distribution in the second batch of detected samples.

**Figure 9:**
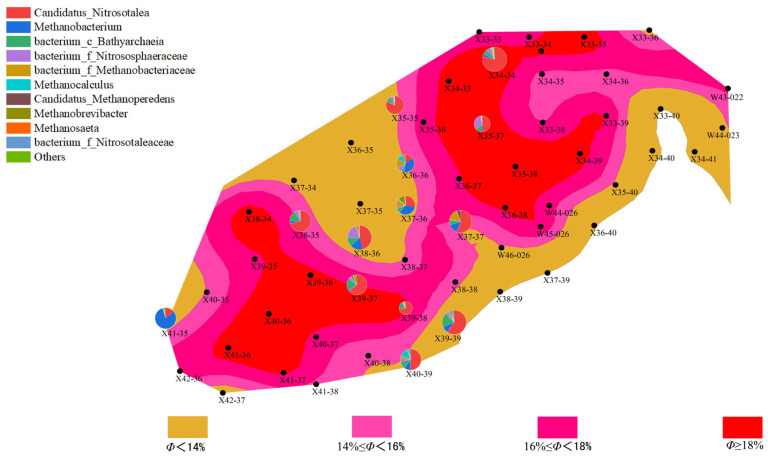
Overlay diagram of reservoir porosity and archaea distribution in the second batch of detected samples.

The distribution of microbial communities is closely related to the reservoir’s porosity and permeability. Well points with higher porosity (“New 39-39” and “New 40-39”) generally exhibited higher microbial activity, especially in the second batch, where archaeal abundance increased. This phenomenon could be due to increased porosity improving the efficiency of organic matter degradation, thus promoting microbial growth and metabolism. In contrast, well points with lower porosity showed lower microbial activity, with both bacterial and archaeal communities having lower abundance. This may be due to limited water and organic matter flow in areas with lower porosity, which affects microbial metabolism and reproduction (Figure 6, Figure 7, Figure 8 and Figure 9).

**Figure 10:**
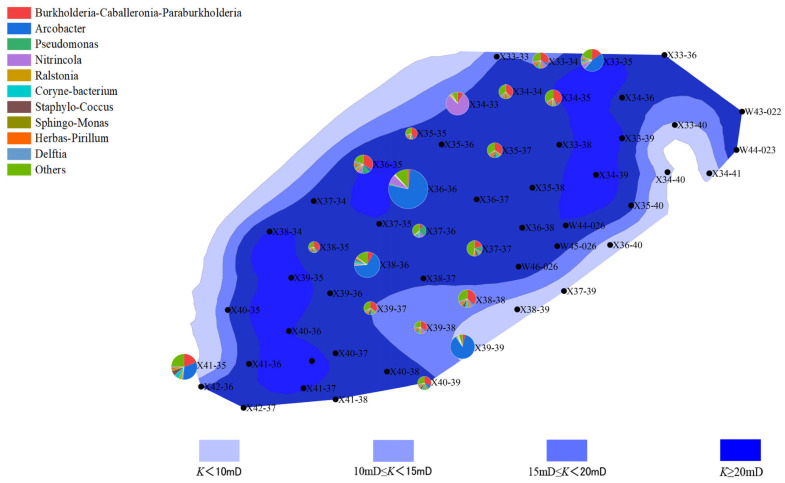
Overlay diagram of reservoir permeability and bacteria distribution in the first batch of detected samples.

**Figure 11:**
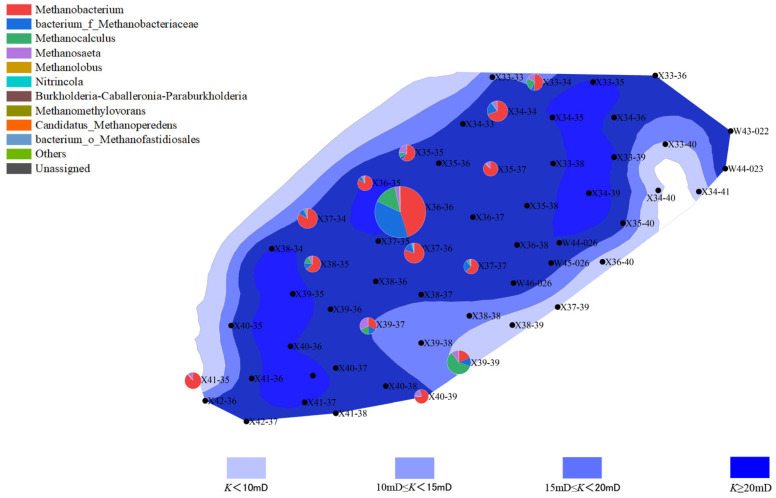
Overlay diagram of reservoir permeability and archaea distribution in the first batch of detected samples.

**Figure 12:**
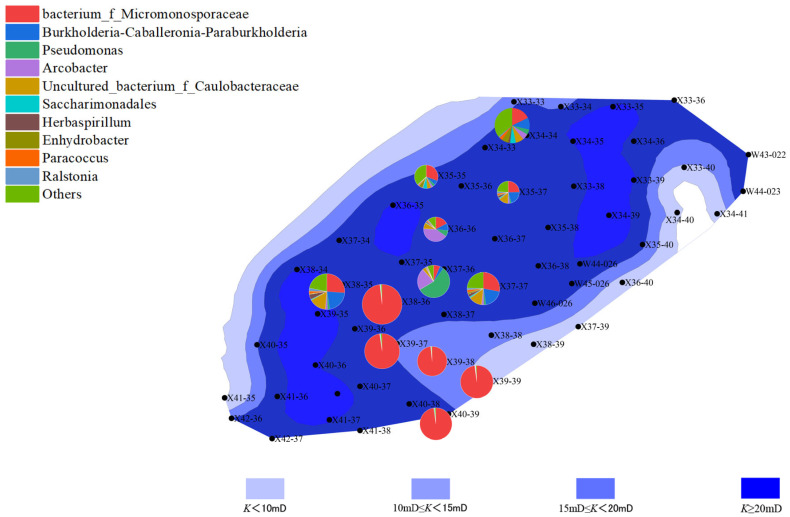
Overlay diagram of reservoir permeability and bacteria distribution in the second batch of detected samples.

**Figure 13:**
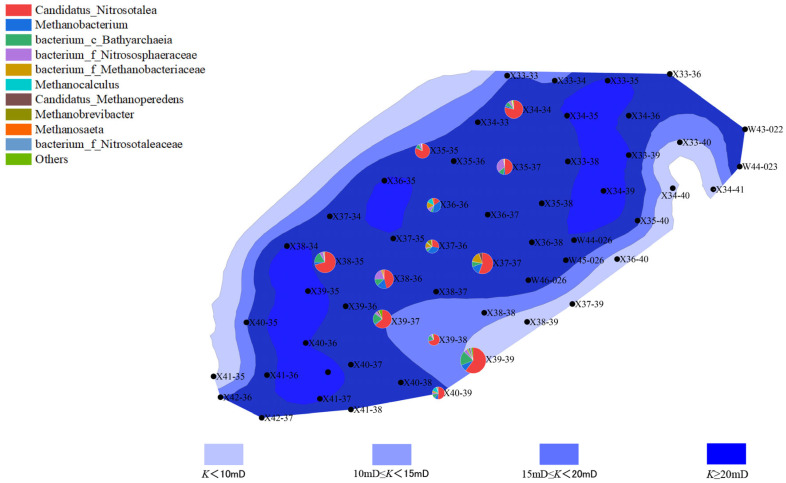
Overlay diagram of reservoir permeability and archaea distribution in the second batch of detected samples.

Well points with higher permeability also generally showed better microbial community performance. Higher permeability facilitates the flow of water, creating a more ideal environment for microbial growth, especially during water flooding, where the activity of both bacteria and archaea is enhanced through more permeable channels. On the other hand, well points with lower permeability, where water cannot effectively penetrate the deeper parts of the reservoir, experienced limited microbial activity, leading to inhibited growth of both bacterial and archaeal communities (Figure 10, Figure 11, Figure 12 and Figure 13).

## 4. Discussion

### 4.1. Microbial Growth Status (Concentration) and On-Site Effects

In the detection of microbial concentrations in two batches, the microbial concentrations in all three propagation pools increased, indicating that microorganisms can reproduce and survive normally in low permeability underground reservoirs. Microbial enhanced oil recovery (MEOR) technology is feasible for the oil displacement process in low permeability reservoirs, and the increase in microbial concentration has positive effects. Zhi’s research shows that, despite the complex reservoir environment, certain microbial strains exhibit good adaptability in low permeability reservoirs and can survive and reproduce under different geological conditions [49]. Yin pointed out that by introducing functional microorganisms, it is possible to trigger an alternative stable state of the reservoir microbial community, thereby promoting the sustainability of MEOR technology [50]. The research findings are consistent.

Preferentially select 33 water injection wells and 95 production wells in the main development area of Block 169 to conduct field trials, covering petroleum geological reserves of 547.4 × 10^4^ t and an annual oil production scale of 4.0 × 10^4^ t/a. The scheme is designed as follows: add microorganisms and nutrients to the propagation device and inject them into the oil layer through the surface water injection system. The designed injection volume is 0.2 times the pore volume, and the injection cycle is approximately 9 years. Currently, the daily microbial injection volume is 450 m^3^/d, the daily actual injection volume is 379 m^3^/d, and the daily nutrient agent injection volume is 600 kg/d, with a mass fraction of the nutrient agent of 0.13%. Within 3 months of injection, the overall reonal dynamics remain stable, with stable water content, and subsequently, multiple production wells have shown initial effects [51,52]. Ghazali Abd. Karim et al. (2001) introduced the application of MEOR technology in the Bokor oilfield in Sarawak, Malaysia [53]; Town et al. (2010) reported the successful application of MEOR in southern Saskatchewan, Canada [54]; Strappa et al. (2004) described a successful MEOR pilot project in the Vizcacheras oilfield in Argentina [55].

### 4.2. Microbial Metabolites and Changes

Satyajit Chowdhury pointed out that lipids have the ability to emulsify crude oil and reduce the interfacial tension between oil and water, including glycolipids and lipopeptides [56]. Acids include short-chain organic acids (such as formic acid, acetic acid, propionic acid, and butyric acid) and long-chain organic acids. Luquot conducted experiments to study the influence of CO_2_ injection on the porosity and permeability of carbonate rocks, demonstrating that acidic fluids can effectively dissolve carbonate rocks, thereby increasing porosity and permeability [57]. Kapse noted that carbon dioxide is one of the important byproducts of microbial metabolism and can enhance oil recovery by increasing formation pressure, reducing crude oil viscosity, and other mechanisms [58]. Javier mentioned that methane is one of the final products of microbial metabolism. Its generation process can increase formation pressure, and the presence of methane can improve the fluidity of crude oil [59]. Hydrogen can be produced by microbial fermentation of hydrocarbons in reservoirs. Moreover, under acidic conditions, the production of hydrogen can stimulate the activity of methanogenic bacteria, thereby converting carbon dioxide into methane and further enhancing oil recovery. Through microbial metabolite monitoring experiments, we found that the aforementioned substances can be produced during the microbial metabolism process. Combined with the research of the above scholars, this confirms their promoting effects on the oil displacement process.

The reduction in the concentration of secondary metabolites is primarily attributed to the dynamic response of the microbial community and the accumulation of environmental pressures: as microorganisms transition from the logarithmic growth phase to the stationary phase, energy allocation shifts from product synthesis to cell maintenance; the accumulation of organic acids leads to a decrease in environmental pH, which inhibits the activity of acid-producing bacteria while activating methanogens. The latter consume organic acids to produce CH_4_. Additionally, the depletion of carbon sources and intensified interspecies competition further weaken the ability to generate metabolic products. The decline in metabolite concentration suggests the need for dynamic adjustment of MEOR (microbial enhanced oil recovery) strategies: supplementing carbon sources in phases and injecting buffering agents to maintain the activity of acid-producing bacteria; introducing acid-tolerant functional bacteria to compensate for insufficient surfactant production; and implementing real-time monitoring of community structure and metabolic gene abundance to provide early warnings of metabolic pathway imbalances, ensuring the sustainability and stability of oil displacement effects [60,61].

### 4.3. Distribution Patterns of Microbial Communities

Fluorescence quantitative PCR and high-throughput gene sequencing technologies have revealed the dynamic changes in the abundance of functional genes such as bacteria, archaea, and methanogens. These changes are closely related to geological characteristics of reservoirs, such as permeability and injection-production layer positions. The reasons for the analysis of gene abundance data are as follows: On one hand, as the bacterial solution is injected into the formation, various bacterial strains continuously proliferate in the reservoir environment, leading to an overall increase in the concentration of functional genes. On the other hand, except for a few wells, as the bacterial solution flows through the pore spaces of the reservoir, the functional gene concentrations gradually balance out among the wells, resulting in reduced differences and a trend toward uniformity. This finding is consistent with the research by Liang, who investigated the phenomenon of horizontal gene transfer in microorganisms within reservoirs. Liang pointed out that the flow of bacterial solution through the pore spaces of the reservoir leads to the balancing of functional gene concentrations among different wells [62].

The microbial diversity in the Se 169 reservoir is remarkable. In the extreme environment of the reservoir, characterized by high temperature, high pressure, and high salinity, the direct oil-displacing effects of various microorganisms and the oil-displacing effects of their metabolic byproducts are conducive to improving the recovery rate of the block. Liu’s research has provided strong evidence for this. In low permeability reservoirs, the microbial communities exhibit strong heterogeneity [63]. The differences in the distribution of microbial communities are related to the local reservoir environment (such as reservoir temperature, pressure, depth, porosity, and permeability). Through the overlay map of reservoir porosity, permeability, and microbial genus distribution, it can be observed that wells with higher porosity and permeability tend to have higher microbial activity. Therefore, when implementing microbial flooding in reservoirs with strong heterogeneity, it is necessary to ensure good connectivity between injection and production wells and around the production wells, as well as to enhance the permeability of the reservoir.

### 4.4. Important Findings and Limitations

This study confirms that microbial activated water flooding also has a promoting effect on reservoirs with an average permeability far lower than the recommended 50 mD. Additionally, the relationship between the heterogeneity of microbial communities and reservoir geological conditions was discovered, providing an important theoretical basis for the layer and well selection, as well as the optimization of injection and production schemes, for microbial enhanced oil recovery in low permeability and heterogeneous reservoirs.

However, the current study has certain limitations. Although encouraging results have been observed in laboratory research and preliminary field tests, the specific concentration change patterns of different microbial metabolites in the process of microbial enhanced oil recovery (MEOR) remain unclear. These metabolites are diverse, including short-chain and long-chain organic acids, lipids, alcohols, ketones, and biogas, all of which play a crucial role in enhancing oil recovery. However, the precise mechanisms of their production, consumption, and mutual transformation in complex reservoir environments are still largely unknown.

Moreover, the long-term effectiveness and sustainability of the field application of MEOR face significant challenges and require further research. Although preliminary field tests have demonstrated the potential of MEOR to enhance oil recovery, maintaining this effectiveness over a long period is crucial for its commercial feasibility. Factors such as microbial population dynamics, nutrient supply, and reservoir heterogeneity can all affect the long-term stability of the MEOR process. For example, the depletion of nutrients or the accumulation of metabolic by-products may inhibit microbial activity, leading to a decline in oil recovery efficiency over time. In addition, the adaptability and evolutionary potential of microorganisms in the reservoir environment will either enhance or weaken the effect of MEOR depending on specific conditions and the microbial strains involved.

## 5. Conclusions

Microorganisms can reproduce and survive normally in low permeability underground reservoirs, making microbial enhanced oil recovery (MEOR) technology feasible for promoting the oil displacement process in low permeability reservoirs. The metabolic byproducts of microbial activation water flooding in low permeability reservoirs mainly include a large amount of lipid surfactants, organic acids, and a small amount of biogas. Microbial flooding can enhance oil recovery efficiency. During the application of microbial flooding technology, it is necessary to dynamically adjust the MEOR strategy to reduce the impact of decreased microbial product concentrations on oil displacement efficiency.

As microbial activation water is continuously injected, the gene counts of bacteria, archaea, and methanogens in the production well fluids generally increase, and the differences in microbial concentration distribution among wells gradually decrease. The microbial communities in low permeability reservoirs exhibit strong heterogeneity, and the variability in microbial community distribution is related to local reservoir conditions, such as temperature, pressure, depth, porosity, and permeability.

Overall, as a low-cost and environmentally friendly technology, microbial flooding has broad application prospects in the development of low permeability reservoirs.

## Figures and Tables

**Table 1:** Primers used for gene detection.

Gene Name	Primer Name	Primer Sequence	Length of PCR Product
16S rRNA	338F	ACTCCTACGGGAGGCAGCAG	480 bp
	806R	GGACTACHVGGGTWTCTAAT	
ARCH	524F_10_ext	TGYCAGCCGCCGCGGTAA	350 bp
	Arch958R_mod	YCCGGCGTTGAVTCCAATT	
mcrA	MLf	GGTGGTGTMGGATTCACACARTAYGCWACAGC	476 bp
	MLr	TTCATTGCRTAGTTWGGRTAGTT	

**Table 2:** Concentration data of metabolites in different experimental batches.

Metabolite Types	Classification	Batch 1			Batch 2		
		Pool 1	Pool 2	Pool 3	Pool 1	Pool 2	Pool 3
Short-Chain Organic Acids (mg/L)	Formic Acid	-	16	8	-	4	2
	Acetic Acid	97	111	117	24	63	32
	Propionic Acid	-	-	-	3	7	2
	Butyric Acid	4	6	5	3	8	3
	Valeric Acid	6	-	-	6	-	-
	Total	108	133	130	36	82	39
Long-Chain Organic Acids (mg/L)	-	0.34	0.61	1.1	0.3	0.86	0.43
Surfactants (mg/L)	Lipids	4.98	1.86	1.57	1.52	1.33	6.06
Organic Solvents (mg/L)	Alcohols	2.66	2.81	1.74	0.06	3.12	0
	Ketones	2.48	4.12	2.55	0.81	1.94	3.85
	Aldehydes	0	1.22	1.24	0.12	0.30	0.60
	Ethers	1.73	0	1.77	0.11	0.51	0
	Total	6.87	8.15	7.3	1.1	5.77	4.55
Biogas (mL/L)	Carbon Dioxide	16	33	31	2	4	2
	Ethane	2	3	3	2	5	1
	Propane	-	2	4	-	2	1
	Total	18	38	38	4	11	4

## Data Availability

The original contributions presented in this study are included in the article. Further inquiries can be directed to the corresponding author.
